# A Card

**Published:** 1864-09

**Authors:** William A. Hammond

**Affiliations:** Washington


A CARD.

The undersigned has read in the *Sunday Morning Chronicle* of this city, the remarks of Judge-Advocate-General Holt on the proceedings of the Court-martial in his case.

He learns from this review and from the order of the President appended, that he has been dismissed the army and prohibited forever from holding office under the United States.

The undersigned has no idea that he will lose one friend by this action of the Administration, but his good name is valuable to him, not only as regards those who know him, but those who do not.

So soon, therefore, as he is furnished with a copy of the findings and sentence of the Court, he will present to the public a brief history of the facts leading to his arrest and trial, a review of the record in his case, and some commentaries on the report of the Judge-Advocate-General.

With these he will be content to submit to the judgment of the world as to how far he has been guilty of the offences charged, and how far he has been the victim of conspiracy, false swearing, and a malignant abuse of official power. WILLIAM A. HAMMOND.

*Washington, Aug.* 22, 1864.

